# Dairy Propionibacteria: Versatile Probiotics

**DOI:** 10.3390/microorganisms5020024

**Published:** 2017-05-13

**Authors:** Houem Rabah, Fillipe Luiz Rosa do Carmo, Gwénaël Jan

**Affiliations:** 1 UMR 1253 Science et Technologie du Lait et de l’Œuf (STLO), Agrocampus Ouest, INRA, F-35042 Rennes, France; houem.rabah@inra.fr (H.R.); fillipelrc@gmail.com (F.L.R.d.C.); 2 Pôle Agronomique Ouest, Régions Bretagne et Pays de la Loire, F-35042 Rennes, France; 3 Instituto de Ciências Biológicas, Universidade Federal de Minas Gerais (ICB/UFMG), 31270-901 Belo Horizonte, Brazil

**Keywords:** dairy propionibacteria, probiotic, metabolites, inflammation, gut microbiota, delivery vehicle, functional food, fermented food

## Abstract

Dairy propionibacteria are used as cheese ripening starters, as biopreservative and as beneficial additives, in the food industry. The main species, *Propionibacterium freudenreichii*, is known as GRAS (Generally Recognized As Safe, USA, FDA). In addition to another dairy species, *Propionibacterium acidipropionici,* they are included in QPS (Qualified Presumption of Safety) list. Additional to their well-known technological application, dairy propionibacteria increasingly attract attention for their promising probiotic properties. The purpose of this review is to summarize the probiotic characteristics of dairy propionibacteria reported by the updated literature. Indeed, they meet the selection criteria for probiotic bacteria, such as the ability to endure digestive stressing conditions and to adhere to intestinal epithelial cells. This is a prerequisite to bacterial persistence within the gut. The reported beneficial effects are ranked according to property’s type: microbiota modulation, immunomodulation, and cancer modulation. The proposed molecular mechanisms are discussed. Dairy propionibacteria are described as producers of nutraceuticals and beneficial metabolites that are responsible for their versatile probiotic attributes include short chain fatty acids (SCFAs), conjugated fatty acids, surface proteins, and 1,4-dihydroxy-2-naphtoic acid (DHNA). These metabolites possess beneficial properties and their production depends on the strain and on the growth medium. The choice of the fermented food matrix may thus determine the probiotic properties of the ingested product. This review approaches dairy propionibacteria, with an interest in both technological abilities and probiotic attributes.

## 1. Introduction

Propionibacteria are high-GC content, non-motile, non-spore forming, anaerobic to aerotolerant, gram-positive bacteria, which belong to the actinomycetales order. They are therefore highly distinct from low GC content firmicutes, which include lactic acid bacteria. Actinomycetales comprise bacterial species with a mycelium-like aspect, found in various environments, including animal hosts and soil, and are known for a prolific production of small molecules, including antimicrobials. Propionibacteria morphology is described as pleomorphic rods, or small cocci, arranged in pairs, short chains or clusters resembling “Chinese characters” [1].

**Figure 1:**
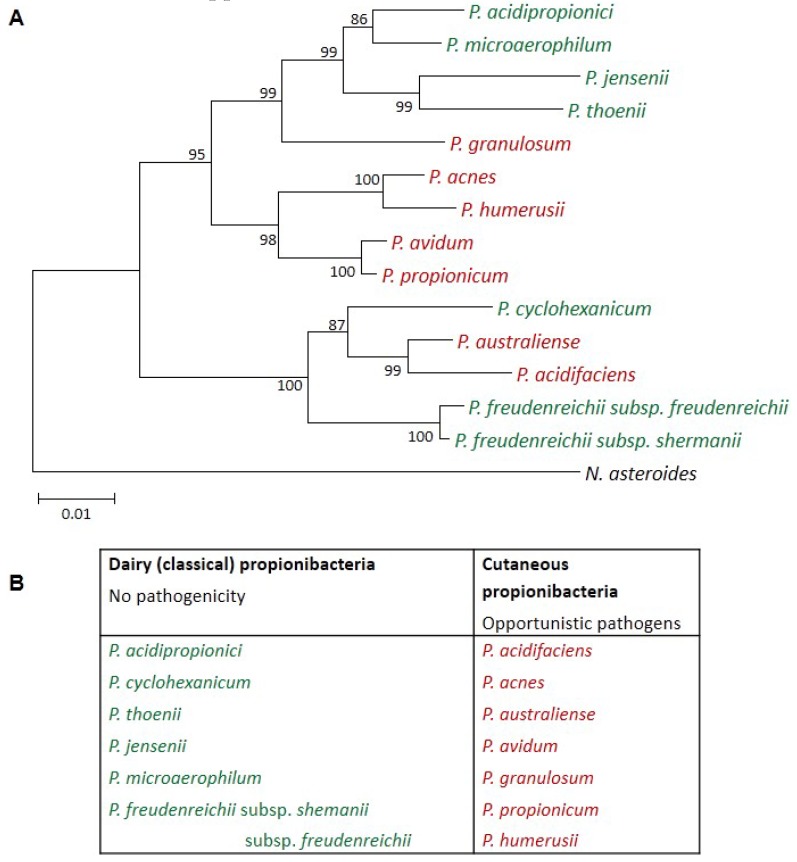
(**A**) Propionibacteria minimum evolution phylogenetic tree based on 16S rDNA sequences. The 16S rDNA sequence of the Actinomycetale *Nocardia asteroides* was used as a distant outgroup to root the tree. Adapted from McDowell et al. [2]; (**B**) Repartition of *Propionibacterium* species in two distinct groups. The species formerly known as *P. inoccuum* and *P. lymphophilum* have been reclassified as *Propioniferax innocua* and *Propionimicrobium lymphophilum* respectively. *P. freudenreichii* received the GRAS (generally recognized as safe) status. Adapted from Cousin et al. [5]. Dairy species are presented in green and cutaneous ones in red.

The genus *Propionibacterium* comprises both cutaneous species (including the well-known *P. acnes*), which may act as opportunistic pathogens, and dairy species, which have no reported adverse effects. Figure 1 shows the different species as well as their phylogenetic repartition as described by McDowell et al. (2013) [2]. The dairy species *Propionibacterium freudenreichii* and *Propionibacterium* acidipropionici are clearly distinct from cutaneous species. *P. freudenreichii* has the GRAS (Generally Recognized As Safe) status in accordance with a long and documented history of safe use in food [3]. *P. freudenreichii* is widely cultivated and consumed by humans in fermented dairy products such as Swiss-type cheese and in food probiotic supplements. *P. freudenreichii* and *P. acidipropionici* have also been listed in the QPS (Qualified Presumption of Safety) list by the European food safety authority [3]. More generally, dairy propionibacteria have attracted attention as potent probiotics. A probiotic is defined as “a live microorganism which, when administrated in adequate amount, confers a health benefit on the host” [4]. Recent data also suggest the ability of some dairy propionibacteria metabolites to be used as prebiotics [5,6], such as 1,4-dihydroxy-2-naphtoic acid (DHNA): a selectively fermented ingredient that allows specific changes, both in the composition and/or activity in the gastrointestinal microflora that confer benefits [7,8,9,10,11,12,13].

Genome sequencing of P. freudenreichii and P. acidipropionici revealed the genetic basis of their great ability to adapt to various environments [14,15]. Moreover, they display a peculiar fermentative metabolism, which relies on propionic fermentation and may use various carbon and energy sources, releasing in the extracellular medium various beneficial metabolites. Recently, an accumulation of promising data, both in vitro and in vivo, evidenced a strong potential as probiotic bacteria in food, able to modulate beneficially the gut microbiota, metabolism, physiology and immunity through valuable metabolites [5]. This review will thus focus on their beneficial effects, their molecular mechanism of action and their applications.

## 2. Gut Persistence of Dairy Propionibacteria

### 2.1. Digestive Stress Tolerance

The Gastrointestinal tract is a complex ecosystem where physicochemical environment is unfavorable to exogenous microorganisms. A probiotic microorganism must be able to persist in the host gut, to deliver and produce beneficial metabolites. Therefore, tolerance to digestive stresses is one of the main factors limiting the use of microorganisms as live probiotic agent [16,17].

Gastric acid and bile salts are defense mechanisms encountered during intestinal transit whereas pancreatic secretions can also exert some antimicrobial activity via digestive enzymes. Dairy propionibacteria are particularly hardy and robust, compared to other probiotics, which is in accordance with their ecology. They show high tolerance in vitro to stimulated human upper gastrointestinal tract conditions, depending on species and strain type. The growth or delivery medium may also provide protection [18,19,20,21,22,23,24]. The tolerance response results in various modifications such as morphological changes or proteins expression. During exposure to acid and bile salts, *P. freudenreichii* expresses general stress proteins and induces regulatory genes involved in cellular response to membrane perturbation, oxidative stress and DNA damage [25,26,27,28]. *P. acidipropionici* showed the same high tolerance response to acid stress [29,30]. Microbiota competition for nutrients constitutes also a limiting factor for dairy propionibacteria persistence in the gut. However, dairy propionibacteria are able to metabolize various carbon and nitrogen sources, to produce reserve compounds such as polyphosphate, glycogen and trehalose, which also have an osmoprotectant role [22,31]. These results were reinforced by in vivo studies; *P. freudenreichii* was shown to maintain a metabolic activity, in addition to survival, within the human and animal digestive tracts [9,32,33,34,35]. Indeed, *P. freudenreichii* orients its genome expression towards the use of intestinally available substrates such as propanediol, gluconate and lactate, to sustain its metabolism, thus avoiding starvation during digestive transit [35]. Their concentration reached the adequate bacterial amount in the gut for probiotic applications.

### 2.2. Adhesion to the Gut Epithelium

**Figure 2:**
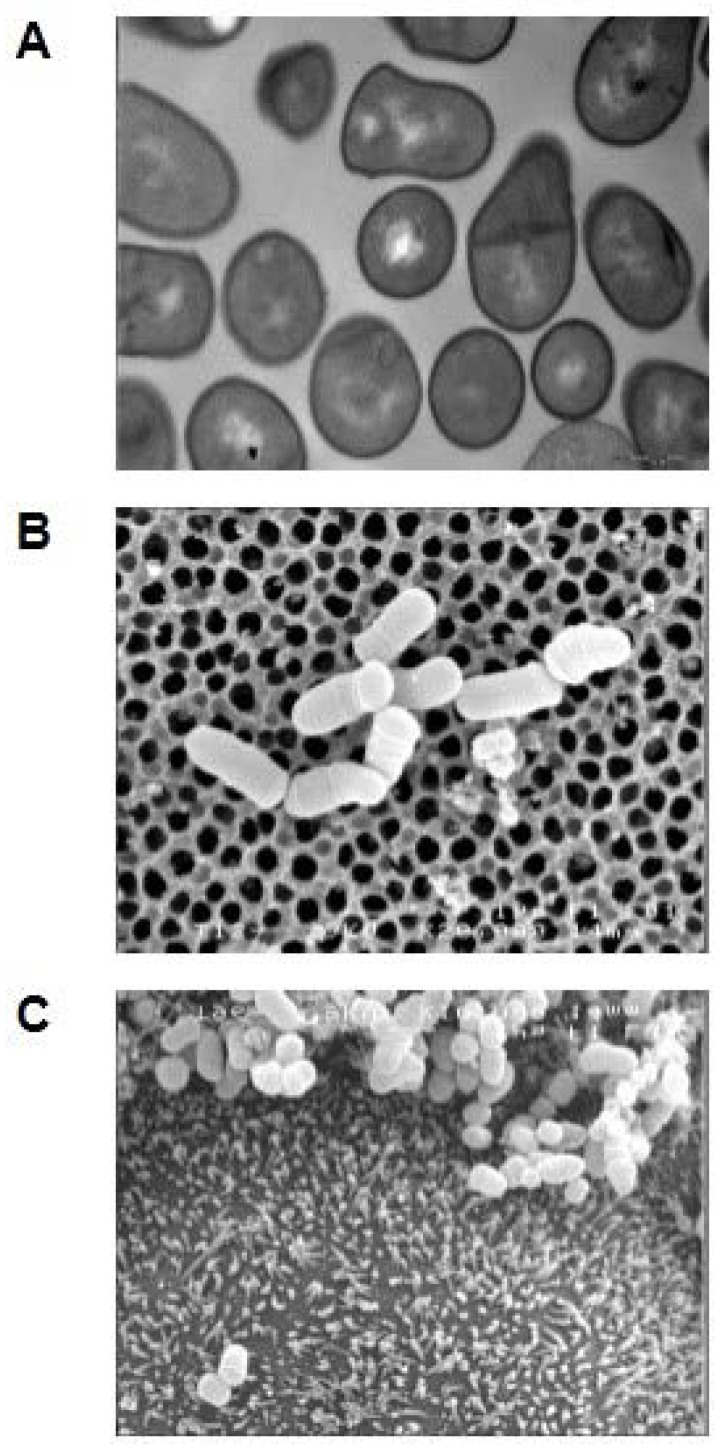
Electron microscopy analysis of *Propionibacterium freudenreichii*. The Propionibacteria were cultivated alone (**A**,**B**) or in contact with cultured human intestinal cell line Caco2 (**C**). Observation was made using either transmission (**A**) [46] or scanning (**B**) [26] and (**C**) (personal communication), electron microscopy.

Besides the ability to withstand digestive stresses, probiotic microorganisms should persist in the digestive tract to interact with host cells and exert their expected beneficial effects. Probiotics lifespan in the digestive tract will depend on their capacity to adhere to intestinal mucosa and on their growth rate. Propionibacteria species have a slow growth rate, so that adhesion and adaptation constitute the bottleneck of their beneficial effects within the host. Numerous studies showed the capacity of *P. acidipropionici* and *P. freudenreichii* to adhere to human and animal intestinal cells [19,36,37], as well as to human and animal intestinal mucus [38,39,40]. Nonetheless, the adhesion rate evaluated in vitro varied between 0.03 to about 40%, depending on many factors as adhesion model used (cells or mucus), species types, strain type and growth or vehicle medium [19,23,24,38,39,40]. Figure 2 shows physical interaction of dairy propionibacteria with cultured human colon epithelial cells. *P. freudenreichii* adhesion was evidenced even after heat inactivation [38]. Propionibacteria adhesion to intestinal cells leads to exclusion of invasive pathogenic bacteria by competitive adhesion or co-aggregation mechanisms. This concerns pathogens such as *Escherichia coli*, *Pseudomonas aeruginosa*, S*taphylococcus aureus* and *Salmonella enterica* [41,42,43,44]. Adhesion mechanisms remain poorly understood, but some preliminary experiments point out a role of surface proteins and teichoic acid in adhesion mechanisms [37]. Recently, a surface proteome study in *P. freudenreichii* evidenced the presence of two conserved proteins known to be involved in adhesion, in other bacterial species [45]. The first protein is Internalin A, which has Leucine Rich Repeat domains (LRR) known to be involved in protein/protein interaction. The second is BopA, belonging to the ABC superfamily, with an ATP binding cassette, showing homologies to a bifidobacteria adhesion protein. However, other components secreted may play a role in adhesion. Indeed *P. freudenreichii* was shown to secrete a lipopeptide having biosurfactant properties and an anti-adhesive effect on *P. aeruginosa* [43]. All these studies suggest the ability of dairy propionibacteria to adhere to the intestinal mucosa, allowing them to persist in the host. However, no in vivo analysis exist to assess specifically dairy propionibacteria adhesion, nonetheless some studies in animals and humans suggest there is only a transient colonization, since fecal propionibacteria population in human volunteers decreases after ceasing the ingestion of propionibacteria [34]. The beneficial effect of the promising metabolites produced by dairy propionibacteria would certainly be favored by their ability to tolerate digestive stresses and to adhere to epithelial cells, allowing close contact of the probiotic and the target cells.

## 3. Microbiota Modulation by Dairy Propionibacteria

The gut microbiota plays a role in several aspects of the host physiology, including metabolism, defense against pathogens, immune system maturation and brain development. An imbalance of the microbiota composition could be an initiator or a consequence of the development of much pathology, such as inflammatory diseases, colorectal cancer or *Clostridium difficile* infection. In disease contexts such as Inflammatory Bowel disease (IBD), patients present a lower microbiota diversity, which could initiate or exacerbate inflammatory disease [47]. Microbiota manipulation by fecal microbiota transplantation, prebiotic or probiotic consumption is a promising way to improve or to restore the microbiota diversity [47]. Modulation of the gut microbiota in animals and human being, as a result of *P. freudenreichii* and *P. acidipropionici* consumption, was reported in the context of colitis [7,13,48,49] and in healthy subjects [8,50,51,52]. These studies reported an increase in the genus of Bifidobacteria, which are well known for their positive health benefits to their host via their metabolic activities [53]. Dairy propionibacteria were also shown to decrease Bacteroides genus which possess an enterotoxin associated with the prevalence of IBD [54], and Clostridium genus, strains of which are associated with severe intestinal infections [47]. Modulation of the gut microbiota to favor symbiotic bacteria such as Bifidobacteria, and at the expense of opportunistic pathogens, is not fully understood. However, the bifidogenic effects described for dairy propionibacteria were attributed to the release of two small molecules, 1,4-dihydroxy-2-naphtoic acid (DHNA) and 2-amino-3-carboxy-1,4-naphthoquinone (ACNQ) [55]. DHNA is a vitamin K2 (or menaquinone) biosynthesis intermediate [56]. DHNA treatment was shown to restore *Lactobacillus* and *Enterobacteriacea* flora in dextran sulfate sodium (DSS)-induced-colitis in mice [13]. In addition, it induces expression of the anti-microbial C-type lectin Reg III protein family, which certainly affect microbial flora [48]. Elsewhere, ACNQ enhances the activity of NADH peroxidase and NADH oxidase in Bifidobacteria. It serves as an electron acceptor of NAD(P)H diaphorase and as an electron donor of NAD(P)H peroxidase [49,57,58]. Regeneration of these cofactors in Bifidobacteria is reported to enhance their growth. Indeed, consumption of dried cultures of the *P. freudenreichii* ET-3 strain, provided by the Japanese company Meiji, led to an enhanced population of Bifidobacteria within the human gut microbiota in healthy male and female human volunteers [50]. Similar modulation was obtained using a cell-free culture supernatant of *P. freudenreichii*, which was called bifidogenic growth stimulator (BGS), attesting the role of secreted components in the bifidogenic effects [49,51,57,59]. BGS was tested in humans at high doses, up to 45 tablets daily, without noticeable adverse effect, showing the potential and the safety of use of those components as prebiotics [60]. The use of dairy propionibacteria as an animal probiotic to modulate gut microbiota or pathogen infections is also a promising new application of dairy propionibacteria. Indeed, interesting results were obtained using *P. acidipropionici*, by slowing colonization by Bacteroides in the early stage of rearing chicks [52]. The presence of *P. acidipropionici* also limited the growth of *Bacteroidetes fragilis* and *Clostridium hystoliticum* groups in mice cecal slurries with and without fiber supplementation [61]. In addition, several strains of dairy propionibacteria were able to inhibit in vitro *Steptococcus bovis* in ruminal acidosis context [62].

## 4. Immunomodulation by Dairy Propionibacteria

Inflammatory diseases, such as inflammatory bowel disease (IBD), allergy, asthma or rheumatoid arthritis, are a public health problem and affect mainly the developed countries. These diseases are complex, and their precise etiology remains poorly understood. Risk factors related to the immune system, environment, genotype and especially the intestinal microbiota, seem to be involved. There is increasing indication of the potential of probiotics consumption, as a supplement to treatment, to limit the occurrence of some inflammatory diseases [63]. The present section focuses on immunomodulation by dairy propionibacteria in the context of IBD. However, clinical studies have demonstrated beneficial effects of dairy propionibacteria, in combination with other probiotic bacteria, to positively modulate the immune system.

IBD includes two main pathologies: ulcerative colitis (UC) and Crohn’s disease (CD). They are thought to result from an abnormality of the immune response of the intestine with respect to certain components of the intestinal flora occurring in genetically predisposed individuals. There are arguments indicating that consumption of selected strains of probiotic microorganisms could play a favorable role in the treatment of UC [64,65]. In vitro and in vivo data suggest the ability of dairy propionibacteria, specifically *P. freudenreichii*, to modulate the gut immune system and alleviate the inflammation in the context of inflammatory bowel disease. In conventional mice, trinitrobenzene sulfonic acid (TNBS)-induced colitis was prevented by the consumption of *P. freudenreichii* in a strain-dependent manner. Strains inducing high levels of the regulatory cytokine interleukin 10 (IL10) in human peripheral blood mononuclear cells (PBMCs) were the most effective at alleviating TNBS-induced-colitis [66,67]. Immunomodulation exerted by selected strains of *P. freudenreichii* was further evidenced in pigs, with a decrease in plasma haptoglobulin and proinflammatory cytokines as IL-8 and tumor necrosis factor-α (TNFα) in gut mucosa, after lipopolysaccharides (LPS) stimulation ex vivo [8]. Recently, a probiotic mixture containing both *Lactobacillus rhamnosus* and *P. freudenreichii* was tested in humanized mice consuming a high-fat diet. It tended to down-regulate both intestinal and systemic pro-inflammatory changes induced by the diet [68]. When tested in irritable bowel syndrome patients (IBS), it alleviated the symptoms of IBS and stabilized the gut microbiota [69]. In a pilot study, patients with active ulcerative colitis receiving BGS experienced an improvement of the clinical activity index score [49,70]. Dairy propionibacteria exert anti-inflammatory effects through different components that seem to trigger different molecular mechanisms.

### 4.1. Surface Layer Proteins

Different propionibacteria compounds were reported for potential anti-inflammatory effects, including surface proteins called S-layer proteins (Slps). They form a surface-exposed proteinaceous network, which is present in many Gram-positive bacteria other than propionibacteria, as well as in archaebacteria [71]. Slps proteins are non-covalently anchored to the cell wall via S-layer homology domains (SLH). In *P. freudenreichii*, the annotation of the genome revealed the presence of seven genes encoding putative Slps proteins [14]. However, only three Slps proteins were identified by proteomic analysis (SlpA, SlpB and SlpE).The identified internalin A (InlA) also has SLH domains but is not considered as an Slp [45]. The family of genes encoding the Slps proteins exhibits a wide variety of sequences between species but also within the same species, in accordance with the great functional diversity of these proteins: adhesion, virulence factors, transport of molecules, masking of receptors to phages, and protection against environmental stresses [72,73]. In order to demonstrate the immunomodulatory properties of the *P. freudenreichii* Slps, selective extraction of these proteins by guanidine chloride was carried out. Treatment of PBMCs with this protein mixture induced the release of regulatory interleukin IL-10, in a dose-dependent manner, with little or no secretion of pro-inflammatory factors (IL-12, TNF-α and IL6) [45]. Moreover, this extract, when applied in conjunction with a proinflammatory strain such as *Lactococcus lactis* MG1363, considerably reduces the induction of the proinflammatory cytokines IL-12, IFN-γ and TNF-α by this strain. This confirms that extractible surface proteins modulate the release of immunomodulatory cytokines. In order to identify the immunomodulatory properties of each surface protein, the *P. freudenreichii* CIRM-BIA 129 strain, which has a very marked anti-inflammatory profile, has been mutated for the *slp b and slp e* genes [74]. The mutations induced suppression of the anti-inflammatory effect of this strain on human PBMCs, this property seem to be a result not of the presence of one protein, but of a combination of several surface proteins [74]. Some of the strains of *P. freudenreichii* that fail to modulate the immune response are covered by an extracellular capsule of exopolysaccharides (EPS) [75,76]. Removal of this EPS (by mutational inactivation) unmasks surface proteins and confers immunomodulatory properties to the mutant [75,76]. This indicates a key role of surface proteins as Microbe-Associated Molecular Patterns (MAMPs) in this probiotic/host cross-talk, with promising anti-inflammatory applications. Indeed, as demonstrated for *Lactobacillus acidophilus* S-layer protein A, dairy propionibacteria S layer proteins are supposed to interact with immune cells as dendritic cells via specific receptors [77,78], inducing tolerance response leading to attenuated colonic inflammation.

### 4.2. Short Chain Fatty acids (SCFAs)

Different metabolites known for immune system modulation include the short chain fatty acids (SCFAs). SCFAs are produced mainly in the colon by colonic bacteria. Butyrate (C4), propionate (C3) and acetate (C2) are the major SCFAs produced by fiber or complex carbohydrate fermentation to be used as an energy source by mainly colonocytes and hepatocytes. Dairy propionibacteria produce mainly acetate and propionate in ratio 2:1 by anaerobic fermentation of carbohydrates or organic acids. There is a wealth of published scientific data on the central role of SCFAs in the regulation of the intestinal immune system [79,80,81]. Indeed, SCFAs impact on intestinal immunity will depend on the existing immune environment. Concerning dairy propionibacteria, a transcriptomic analysis of HT29 cells showed a modulation by *P. freudenreichii* or SCFAs treatments of NOD-like receptors and cytokine-cytokine receptor interaction gene expression, known to play a role in immune response [10]. In addition, an HDAC inhibitory activity was highlighted, which demonstrates the potential of dairy propionibacteria to modulate gut inflammation through SCFAs. HDACs inhibition activity degree varies with SCFAs nature (Butyrate > propionate > acetate) [80]. HDAC inhibition seems to be induced in part by SCFAs activated G protein–coupled receptors. Their activation by SCFAs modulates gut inflammation through regulation of activation, proliferation and differentiation of immune and epithelial cells [80,81,82].

### 4.3. Conjugated Fatty Acids

Another promising beneficial metabolic activity is the ability, shared by other probiotics, to convert free linoleic acid (LA, C18:2), α-linolenic (LNA, C18:3), γ-linolenic (GLA, C18:3) and stearidonic acids (SA, C18:4) into their respective conjugated fatty acid (CLA, CLNA, CGLA and CSA) [83,84,85,86]. Conjugated fatty acids (CFAs) are a mixture of a number of geometric and positional isomers of octadecadienoic acids. Until today, there have been a few studies reporting the ability of propionibacteria to produce some CFA isomers [83,84,86,87,88], however their biological effects have not been investigated. CFAs production by dairy propionibacteria is a way to cope with the inhibitory effect of fatty acids on bacterial growth [88]. Dairy propionibacteria, such as *P. freudenreichii*, produce the isomer cis-9, trans-11 octadecadienoic (Rumenic acid, RA) in culture and in fermented dairy products from LA; and isomerize also the c12-double bond of LNA and γ-linolenic acid [84,85,87,89]. Nonetheless, animals and clinical studies report the anti-inflammatory effects of CLA and CLNA including those produced by dairy propionibacteria, at different level of modulation, according to isomer type, by acting as PPARγ agonists. PPARγ (peroxisome proliferator-activated receptor γ) is a nuclear receptor forming obligate heterodimer with retinoid X receptor (RXR). PPARγ activation by CFAs can regulate the expression of its target genes involved in adipogenesis, lipid metabolism, inflammation and maintenance of metabolic homeostasis [90,91]. It also interferes with other proteins and transcription factors such as NF-κB and AP-1 through repression mechanisms [91]. Indeed, CLA and CLNA decrease antigen-induced proinflammatory mediators [92,93,94], modulate immune cells proliferation and differentiation [90,95], and limit adverse effects of colonic inflammation [90,91,95,96]. For dairy propionibacteria, the administration of cheeses matrix containing *P. freudenreichii,* alone or in combination with lactic acid bacteria, was shown to increase *Pparg* mRNA levels in the colon of mice during TNBS-induced colitis [7,12]. This effect could be attributed to the presence of CFAs in fermented cheeses, but additional analysis is required to establish a link between the increase of *Pparg* gene expression and CFA production by dairy propionibacteria.

### 4.4. DHNA

DHNA, described above for its bifidogenic property, exerts an anti-inflammatory effect in different murine colitis models, as murine DSS-colitis and IL10-/- mice that develop spontaneous colitis. DHNA reduces the expression of cell adhesion molecules, as MAdCAM-1 or VCAM-1, depending on colitis model [11,13]. In IBD patients, those adhesion molecules are highly expressed, which aggravates the inflammation by increasing immune cells’ infiltration of tissues. The lymphocyte infiltration observed in experimental mice colitis was clearly diminished by DHNA administration. In addition, DHNA reduces proinflammatory cytokine expression in vivo and in vitro within macrophage cells after endotoxin stimulation [48]. DHNA activate the aryl hydrocarbon receptor (AhR), an important transcriptional factor involved in inflammation. AhR activation seems to be involved in the inhibition of secretion of proinflammatory cytokines. Indeed, the inhibition of proinflammatory cytokine IL6 in LPS-stimulated macrophages was related to AhR activation by DHNA [48].

## 5. Anti-Cancerous Effect

According to WHO, cancers represent a leading cause of morbidity and mortality worldwide, with approximately 8.2 million deaths caused by cancers in 2012. Colorectal cancer, the fourth most common cause of cancer death, is considered to be a Westernized disease with high incidence rates (in North America, Australia, New Zealand and Europe (>40 cases per 100,000). On one hand, significant associations between unhealthy dietary factors and colorectal cancer risk have been demonstrated by several studies [97,98]. On the other hand, gut microbiota appears to govern gut inflammation and colorectal cancer development, specifically through its metabolites. Managing gut microbiota modulation by probiotic and prebiotic consumption may modify gut microbiota metabolism to achieve the objective of preventing colorectal cancer in western societies [97,98].

### 5.1. Short Fatty Acids

Dairy propionibacteria, including strains of *P. acidipropionici*, *P. freudenreichii* subsp *freudenreichii* and *P. freudenreichii* subsp *shermanii,* have been shown to possess the ability to induce apoptosis in colorectal and gastric cancer cells in vitro (HT29, Caco2, and HGT-1 cancerous cell lines) [10,99,100,101] and in vivo within human microbiota associated rats [102]. Propionate and acetate, produced by dairy propionibacteria, were identified as the main actors of this effect [10,99,100,101]. The pro-apoptotic action, exerted on cultured colon cancer cells and confirmed in an animal model of carcinogenesis, was studied in details at the cellular and molecular level. As demonstrated for butyrate, the anti-cancerous effect of propionibacterial SCFA consists in inducing apoptosis. The apoptotic intrinsic pathway is activated: SCFAs act on the mitochondria adenine nucleotide transclactor (ANT) pore, causing mitochondria depolarization and permeabilisation, leakage of cytochrome C and caspase activation [99,100,101]. Furthermore, Cousin et al. (2016) showed that these SCFAs, in combination with TNF-Related Apoptosis-Inducing Ligand (TRAIL) treatment, increased its cytotoxicity, by enhancing TRAIL-R2/DR5 expression in HT29 cells. TRAIL is a death receptor, a tumor necrosis factor receptor superfamily member, which mediates apoptosis by activating the extrinsic apoptotic death pathway [7]. In parallel, such combinations lead to a modulation of genes expression involved in apoptosis, decreasing FLIP_L_ and XIAP expression, which are two apoptosis inhibitors regulating extrinsic and intrinsic cell death pathways, respectively [10]. In addition, inhibition of histone deacetylase (HDAC) in HT29 cells by SCFAs leads to growth inhibition and cell cycle arrest by expression of p21, which was observed at transcriptomic and proteomic levels [10].

### 5.2. Conjugated Fatty Acids

In vitro and in vivo studies showed the anti-proliferative effect of conjugated fatty acids, including those produced by dairy propionibacteria [84,86,87,88,89], on various cancerous cells such as melanoma, colorectal, breast, prostate and hepatoma cell lines [92]. The anti-proliferative kinetic is time and dose dependent. Furthermore, the mechanism and effectiveness vary with the CFAs type—conjugated linolenic acid is more efficient than conjugated linoleic acid—and with isomer type of a particular CFA [93,95]. For some CFAs isomers, the anti-proliferative mechanisms on cancerous cells were characterized at a molecular level: cycle cell arrest, lipid peroxidation and activation of apoptotic pathways are induced by CFA treatment in vitro and in vivo.

Concerning human studies, lower serum levels of CLA in post-menopausal Finnish women was associated with high cancer occurrence, and milk consumption was inversely related to breast cancer risk. In addition, diverse clinical trials suggested the beneficial impact of conjugated fatty acids for prevention or treatment of cancers. As a consequence, biohydrogenation of polyunsaturated fatty acids by probiotic bacteria such as dairy propionibacteria opens the possibility of developing dairy or vegetable fermented food from ingredients rich in polyunsaturated fatty acid, designed for high-risk cancer populations.

### 5.3. Bioremediation against Carcinogenic Components

Dairy propionibacteria, among other probiotic bacteria, have the ability to bind in vitro and in vivo to heavy metals or toxins associated with high risk of cancers, which may contribute to reduced risk factors for cancer development. *P. acidipropionici* showed capacity to remove plant lectins such as concanavalin A and jacalin from colonic cells, which reduced lectins’ toxicity for intestinal cells [103]. Furthermore, selected strains of dairy propionibacteria and of lactic acid bacteria block the intestinal absorption of aflatoxin B1 and remove heavy metals such as cadmium and lead, in a strain-dependent manner [104,105,106,107,108]. The detoxification effect on aflatoxin B1 of dairy propionibacteria was attested by a clinical trial on the population of Southern china continuously exposed to aflatoxin contamination [109].

No clinical evidence on propionibacteria consumption within colorectal cancer (CRC) patients exists, however *P. freudenreichii* uptake by humans increases SCFAs in feces, suggesting the possibility of modulating gut SCFAs concentration with the aim of preventing CRC occurrence. When tested in healthy men, a probiotic mixture containing both lactic acid bacteria and propionibacteria [110,111], led to a reduction in fecal α-glucosidase, which is associated with carcinogenesis.

## 6. Impact of Vectorization on Probiotic Properties

Probiotics are commonly consumed under the form of dried microorganisms, in capsules or tablets. The development of functional foods fermented by dairy propionibacteria is a promising research area. The health benefits of dairy propionibacteria are strain-dependent, but the delivery vehicle also plays a crucial role, which remains barely investigated. Indeed, the matrix affects the metabolites amount or/and the bacterial capacity to persist in the gut. As demonstrated by Cousin et al (2012), the anti-inflammatory effect of *P. freudenreichii* was enhanced upon growth in milk ultrafiltrate medium [9], which could be explained by an enhanced Slp expression compared to a classical growth medium. The biohydrogenation of polyunsaturated acids by dairy propionibacteria may enhance CFAs content in fermented dairy products, but could be limited by the low polyunsaturated acid availability in the matrix. Natural sources of conjugated fatty acids are very limited and are relatively low; the addition of CFA chemically synthesized cannot remedy to some isomers deficiency in foods. Vegetal matrices such as soya, rich in CL and CLN, could be employed to develop new foods enriched in conjugated fatty acids, by dairy propionibacteria fermentation. Similarly, SCFAs production is proportionally related to the amount of fermentable substrates in the medium. Dairy products are naturally rich in lactose, both lactate and lactose can be used by dairy propionibacteria. The delivery vehicle also affects the tolerance response of dairy propionibacteria to digestive stresses and adhesion to cells, depending on its biochemical composition, its physical microstructure and the existing microbial ecosystem [23,24], which directly affect the viable bacterial amount reaching the gut. Growth of dairy propionibacteria on stressful mediums such as fermented dairy products confers a high tolerance to acid and bile salt stresses in vitro and in vivo [20]. In addition, dairy products with a high osmotic pressure enhance storage of trehalose, glycogen and polyphosphate, which could improve nutrients deficiency tolerance in the gut [22]. Some clinical trials confirmed the matrix effect; a probiotic mixture including *P. freudenreichii* was also tested in humans either in conventional capsules, in yogurt or in cheese. The highest fecal quantity of *P. freudenreichii* was yielded by yogurt [112]. Accordingly, yogurt was shown in a French human study to favor not only survival, but also metabolic intestinal activity of *P. freudenreichii* [34].

## 7. Technological Applications of Dairy Propionibacteria

### 7.1. Swiss-Cheese Manufacturing

Dairy propionibacteria, especially *P. freudenreichii*, are mainly employed as a ripening starter for Swiss-type cheese manufacturing, such as Emmental cheese. They contribute to their characteristic flavor and openings [1,113,114]. The openings are due to the production of carbon dioxide (CO_2_), produced during fermentation of lactate and aspartate. These latter are generated in cheese during degradation of lactose and proteins, respectively, by lactic acid bacteria. The ability and the intensity of aspartate metabolism in *P. freudenreichii* are strain-dependent; a high aspartate metabolism leads to a higher proportion of CO_2_. This high aspartate catabolism can be associated with an opening imperfection during Emmental cheese ripening, resulting in the formation of slits and cracks [1,113]. The typical Swiss-type cheese flavor is due mainly to the presence of dairy propionibacteria that produces flavor compounds by three metabolism pathways: lactate and aspartate fermentation, fat hydrolysis, and amino acid catabolism. The lactate and aspartate fermentations lead to the production of organic acids, mainly propionate, succinate, and acetate acid, which are considered to be principal flavor compounds. Free fatty acids are also important for cheese flavor, the lipolytic activity during cheese ripening is mainly due to dairy propionibacteria in strain-dependent manner [113,115]. The third compound is the branched-chain volatile molecules, which are formed from branched-chain amino acid catabolism. The two branched-chain compounds produced by *P. freudenreichii* are 2-methylbutanoic acid and isovaleric acid. In Emmental cheeses, *P. freudenreichii* reaches a high population, with counts over 10^9^ cfu/g of cheese, depending in ripening period. The high tolerance of *P. freudenreichii* to different stresses allows this population to be reached. Indeed, during the cheese manufacturing process, dairy propionibacteria face different stresses, such as high and low temperature, acidification, osmotic stress induced by NaCl; their robustness, compared to the other dairy species, would be responsible for the prevalence of this species in Swiss-type cheeses [113]. Dairy propionibacteria can also be implemented in low amounts in various cheeses without openings to enhance flavor formation [1].

### 7.2. Production of Nutritional Molecules

Dairy propionibacteria produce several nutritional molecules essential to human health such as B vitamins (including cobalamin and folic acid). Indeed, *P. freudenreichii* is the only B12 producer known to be a GRAS bacteria [114]. Vitamin B12 (or cobalamin) is synthesized as a cofactor for propionic acid fermentation. Vitamin B12 is an essential vitamin, required for maintaining healthy nerve cells, for the production of cell’s genetic material and energy, and for other important functions. Vitamin B12 has been industrially produced for a long time by chemical synthesis, which requires more than 70 steps by a chemical method [116]. This production method is too difficult and expensive, compared to the biosynthesis dairy propionibacteria [116,117]. The pathway of vitamin B12 synthesis in *P. freudenreichii* has been completely characterized, and important efforts have been made to improve vitamin B12 biosynthesis by implementing random mutagenesis, genetic engineering and by optimizing fermentation conditions [116,117]. The DHNA, described above as a Vitamin K precursor, has also a potential application as prebiotic to enhance intestinal Bifidobacteria population. There is no industrial production of DHNA, nonetheless some studies investigated how to improve DHNA production by manipulating fermentation conditions [56,114].

### 7.3. Production of Antimicrobial Molecules

*Propionibacterium* spp strains are widely used as food biopreservatives for their antimicrobial activity. They were shown to suppress the growth of mold and undesirable microorganisms in many foods, which prolong their shelf-life [1,6,16,116,117]. Propionic acid is the main anti-microbial molecule produced by dairy propionibacteria. It is commercially available as a Microgard^TM^ product, composed of skim milk fermented by *P. freudenreichii* subsp *shermanii* [116]. The microbial production of propionic acid is limited by parameters including low productivity and low conversion efficiency. However, *P. acidipropionici* species were shown to produce a high amount of propionic acid, by glycerol fermentation, without acetic acid production [117]. Other organic acids are also considered as anti-microbial molecules, including acetic, succinic and lactic acids [116]. In *P. jensenii*, 2-pyrrolidone-5-carboxylic acid, 3-phenyllactic acid, hydroxyphenyl lactic acid and 3-phenyllactic acid were shown to have antimicrobial activity [117]. In addition, different bacteriocins produced by both dairy and cutaneous propionibacteria have been reported and characterized. Bacteriocins are antimicrobial peptides or proteins and are active against other propionibacteria, lactic acid bacteria, other Gram positive bacteria, Gram negative bacteria, yeast and molds. To date, there is no bacteriocin from dairy propionibacteria recognized as GRAS by FDA; more investigation is needed to evaluate their potential application as food biopreservatives or bacteriocin-producer probiotics to inhibit intestinal pathogens.

## 8. Conclusion

The studies reviewed here allowed the development of different tools to screen and elucidate the beneficial properties of dairy propionibacteria strains. Not only the phenotypic traits, but also the molecular bases of probiotic effects are being made available. Dairy propionibacteria are used for various purposes and eaten in various food products. Strains used in the food industry are screened based on technological properties but not on health properties. On the other hand, the technological abilities of probiotic bacteria to produce a fermented food product are rarely studied. Screening of wide collections of propionibacteria for technological and probiotic properties should lead to development of new functional foods. Indeed, specific populations with health problems linked to developed countries life style (intolerance, allergy, inflammation, cancer) will need specific diets. In this context, propionibacteria can play a key role via the modulation of key parameters such as inflammation.
